# A seven-LncRNA signature for prognosis prediction of patients with lung squamous cell carcinoma through tumor immune escape

**DOI:** 10.3389/fonc.2025.1511564

**Published:** 2025-03-24

**Authors:** Qiangqiang Ge, Zhong Lin, Xuequan Wang, Zhengli Jiang, Yan Hu

**Affiliations:** ^1^ Clinical Laboratory, Shangyu People’s Hospital of Shaoxing, Shaoxing, Zhejiang, China; ^2^ Department of Pharmacy, Taizhou Hospital of Zhejiang Province Affiliated to Wenzhou Medical University, Taizhou, Zhejiang, China; ^3^ Department of Radiotherapy, Taizhou Hospital of Zhejiang Province Affiliated to Wenzhou Medical University, Taizhou, Zhejiang, China

**Keywords:** long non-coding RNA, prognosis, lung squamous cell carcinoma, immune escape, seven LncRNA signature

## Abstract

**Background:**

Lung squamous cell carcinoma (LUSC) is a malignant disease associated with poor therapeutic responses and prognosis. Preliminary studies have shown that the dysregulation of long non-coding RNAs (LncRNAs) is linked to cancer development and prognosis. However, research on the role of LncRNAs in LUSC remains limited.

**Methods:**

In this study, we aimed to develop a LncRNA signature for improved prognostic prediction in LUSC and to elucidate the underlying mechanisms. We utilized expression data of LncRNAs and clinical information from 471 LUSC patients in The Cancer Genome Atlas (TCGA), randomly dividing them into a training set (n=236) and a testing set (n=235).

**Results:**

A prognostic signature model comprising seven LncRNAs was constructed using multivariate Cox regression analysis based on the training set. Using a risk score cutoff value of -0.12 (log2-transformed), patients were categorized into high-risk (n=101) and low-risk (n=370) groups. The high-risk group demonstrated significantly worse overall survival (OS) compared to the low-risk group (p<0.0001). The risk score showed strong prognostic predictive ability for LUSC patients, as evidenced by the area under the ROC curve (AUC: 0.66, 0.67, and 0.67) and nomogram analysis (C-index, calibration, and decision curve analysis) for 1-, 3-, and 5-year survival predictions. Independent prognostic factors for LUSC were identified, including risk group (HR=0.3, 95% CI: 0.22–0.4), stage (HR=1.78, 95% CI: 1.28–2.48), and age (HR=1.02, 95% CI: 1.00–1.04). KEGG enrichment analysis revealed that mRNAs influenced by the seven targeted LncRNAs, associated with immune evasion, were primarily linked to pathways such as chemical carcinogenesis, Th17 cell differentiation, NF-κB signaling, and proteoglycans in cancer. Expression levels of 14 target genes related to tumor immune tolerance were significantly suppressed, with eight confirmed via real-time PCR and western blot analysis. Additionally, CIBERSORT analysis of immune cell-related gene expression between normal and LUSC tissues indicated activation of the immune system in LUSC patients.

**Conclusion:**

In conclusion, our findings highlight the clinical significance of the seven LncRNA signature in predicting survival outcomes for LUSC patients.

## Introduction

In recent years, the incidence of lung cancer has been steadily increasing, making it the leading cause of cancer-related deaths worldwide, with a five-year survival rate of less than 15% (1, 2). Despite advances in treatment, the etiology of lung cancer remains largely unclear, and the primary treatment for patients is still surgery combined with adjuvant therapy. The majority of lung cancer cases are classified as non-small cell lung cancer (NSCLC), which primarily consists of lung adenocarcinoma (LUAD) and LUSC. Patients with LUSC are often diagnosed at an advanced stage, limiting the effectiveness of available treatments, which may not be administered in a timely manner. Additionally, LUSC patients generally exhibit lower sensitivity to chemotherapy and radiation compared to patients with small-cell lung cancer. Currently, the tumor node metastasis (TNM) staging system is widely used in clinical settings to guide treatment decisions and predict the prognosis of cancer patients, including those with lung cancer (3, 4). However, the clinical application of TNM staging has certain limitations, such as its inability to accurately predict survival outcomes for many patients following surgical resection, and the presence of inconsistent results among patients within the same stage category (5, 6). Therefore, there is an urgent need to identify novel independent biomarkers for diagnosing and predicting the prognosis of LUSC.

Recent advancements in high-throughput technologies, such as microarrays, sequencing, and mass spectrometry, now allow for the simultaneous evaluation of thousands of molecular expression profiles (7). These breakthroughs, coupled with the growing body of research, have revealed that certain molecular markers are closely associated with tumor phenotype and clinical behavior, particularly LncRNAs. These findings hold significant promise for clinical practice in predicting the long-term outcomes of cancer patients (8–10). Aberrant expression of LncRNAs is frequently observed in various types of cancer and has been linked to tumorigenesis and progression. For example, several well-characterized LncRNAs, such as HOTAIR, MALAT1, and NEAT1, are upregulated in breast cancer, gastric cancer, and hepatocellular carcinoma (11–13). Moreover, multiple differentially expressed LncRNAs have been identified in lung cancer studies, some of which have been implicated in clinical diagnosis and treatment (14). In this study, we identified a set of seven prognostic LncRNA biomarkers associated with overall survival (OS) in LUSC patients. Using these LncRNAs, we developed a 7-LncRNA risk score model that effectively predicts patient OS. These findings were subsequently validated in both the testing set and the entire dataset.

## Materials and methods

### Tissue samples and the reagent

This study included six patients who underwent resection for LUSC at the Department of General Surgery, Taizhou Hospital of Zhejiang Province. All resected specimens were collected and preserved at the Bioresource Center of Taizhou Hospital. The study was approved by the Ethics Committee of Taizhou Hospital.

Reagents used in the study included: Trizol reagent (CW2602M, Beijing Kangwei Century Biotechnology), Reverse Transcription Kit (CW0744M, Beijing Kangwei Century Biotechnology), Fluorescent Quantitative PCR Kit (CW2602M, Beijing Kangwei Century Biotechnology), and primary antibodies including CFLAR, CSF2RA, ICAM1, IL18R1, CISH, CXCL3, IL17D, p-NF-κBα, NF-κBα, and β-Actin (YT0877, YT5262, YT2269, YT5472, YT5920, YT2075, YT6048, YP1372, YT2419, and YT0099 from ImmunoWay Biotechnology).

### Clinical information and RNA expression data

Raw RNA-Seq count data and corresponding clinical information for LUAC patients were obtained from the TCGA-LUAC database (https://xenabrowser.net/datapages/). Patients lacking essential data, such as RNA expression profiles from lung cancer tissue, follow-up survival information, age, gender, and TNM stage, were excluded. Ultimately, 471 patients were included in the study and randomly divided into a training cohort (n = 236) and a testing cohort (n = 235) for model development and validation, respectively. Raw RNA-Seq data were annotated using GENCODE v33 and subsequently normalized using FPKM values.

### Development and validation of the LncRS model

The most significant survival-related LncRNAs were identified using the Least Absolute Shrinkage and Selection Operator (LASSO) regression model in the training group, based on common prognostic LncRNAs previously filtered by univariate Cox regression (P < 0.05). Stepwise multivariate Cox regression analysis was employed to construct the LncRNAs risk signature (LncRS), following collinearity testing based on the Akaike Information Criterion (AIC). The aim was to establish a prognostic signature with optimal predictive capability while using the fewest LncRNAs. The LncRS formula is as follows: LncRS = $\sum_{\left(i=1\right)}^{k}\left(\text{Expi }*\;\beta \text{i}\right)$ , where k and i represent the total number and the sequence number of the significant prognostic LncRNAs, Expi represents the normalized expression values of the corresponding LncRNA for each sample, and βi represents the regression coefficient of the corresponding LncRNA from multivariate Cox regression analysis.

Risk scores were calculated for each patient in the training cohort, and patients were divided into high- and low-risk groups using a predefined cutoff determined by the “survminer” R package. Kaplan-Meier survival curves and log-rank tests were performed to compare survival between high- and low-risk groups. A scatter plot was used to illustrate patient survival status and time based on ascending risk scores, with a heatmap showing the expression levels of LncRS-related LncRNAs. Time-dependent ROC analysis was performed at 1, 3, 5, 7, and 10 years to assess the diagnostic performance of LncRS.

### Independent survival prognostic effect of risk group in TCGA cohort

To assess whether LncRS could serve as an independent prognostic index, univariate (P < 0.2) and multivariate Cox regression analyses were performed, adjusting for clinical factors such as age, gender, tumor location, tumor stage, and pathologic TNM stage in the TCGA cohort (P < 0.05). We also examined the correlation between LncRS-related LncRNA expression levels and survival outcomes. Key findings from univariate and multivariate Cox regression analyses, including Hazard Ratios and P-values, were visualized in a forest plot.

### Nomogram construction and verification

A nomogram was constructed to visually represent the survival probabilities of LUAC patients at 1, 3, and 5 years based on their risk group and key clinical parameters. The concordance index (C-index) and decision curve analysis (DCA) were used to evaluate the prognostic accuracy of the nomogram.

### The prognostic diagnosis accuracy of LncRS verification in TCGA subgroups

Patients in the TCGA-LUAC cohort were stratified into subgroups based on critical clinical parameters, including age, gender, tumor location, tumor stage, and pathologic TNM stage. Kaplan-Meier survival analysis and subgroup forest plots were generated to compare survival between high- and low-risk groups within these subgroups.

### Gene co-expression network and gene functional enrichment analysis

Pearson correlation analysis was used to assess co-expression relationships between LncRS-related LncRNAs and mRNAs in the entire TCGA dataset [correlation coefficient (r) > 0.25, P < 0.05]. Tumor immune-related genes were obtained from GeneCards using the search term “tumor immune” (https://www.genecards.org/). Overlapping genes from these sets were subjected to Gene Ontology (GO) and Kyoto Encyclopedia of Genes and Genomes (KEGG) enrichment analysis. We identified 14 key regulatory genes associated with tumor immune function and pathways through GO and KEGG analysis [r > 0.25; abs (log_2_ Fold change) > 1.3].

### Real-time PCR assay

Total RNA was extracted from LUSC tissue samples using Trizol reagent. The RNA was then reverse transcribed into cDNA using a reverse transcription kit. Quantitative PCR was performed using a fluorescent PCR kit to measure the mRNA expression levels of target genes. Data were expressed as 2^- ΔΔ^Ct, with β-actin serving as the internal control (n=6).

### Western blot assay

Tissue samples were lysed using RIPA buffer containing 1% protease inhibitors in an ice bath for 30 minutes to extract proteins. Protein concentrations were determined using a BCA protein quantification kit (P1511, Beijing Applygen Technologies). Protein samples were separated by SDS-PAGE at 120V for 75 minutes and transferred to PVDF membranes at 250mA. Membranes were blocked with 5% skimmed milk for 2 hours, then incubated overnight at 4°C with primary antibodies (1:1000 dilution). The next day, membranes were incubated with secondary antibodies (1:10,000 dilution) for 2 hours at room temperature, and protein bands were visualized using an ECL kit (P1050, Beijing Applygen Technologies). β-actin served as an internal control.

### Statistical analysis

Statistical analyses were performed using R software (version 4.1.1). Nomogram plots were generated using the “rms” R package. Kaplan-Meier survival analysis was conducted using the “survival” package, with P-values calculated by log-rank tests. Independent prognostic factors were identified through univariate and multivariate Cox regression analyses using the “survival” package.

## Results

### Identification of a prognostic LncRNAs signature in the training set

**Figure 1:**
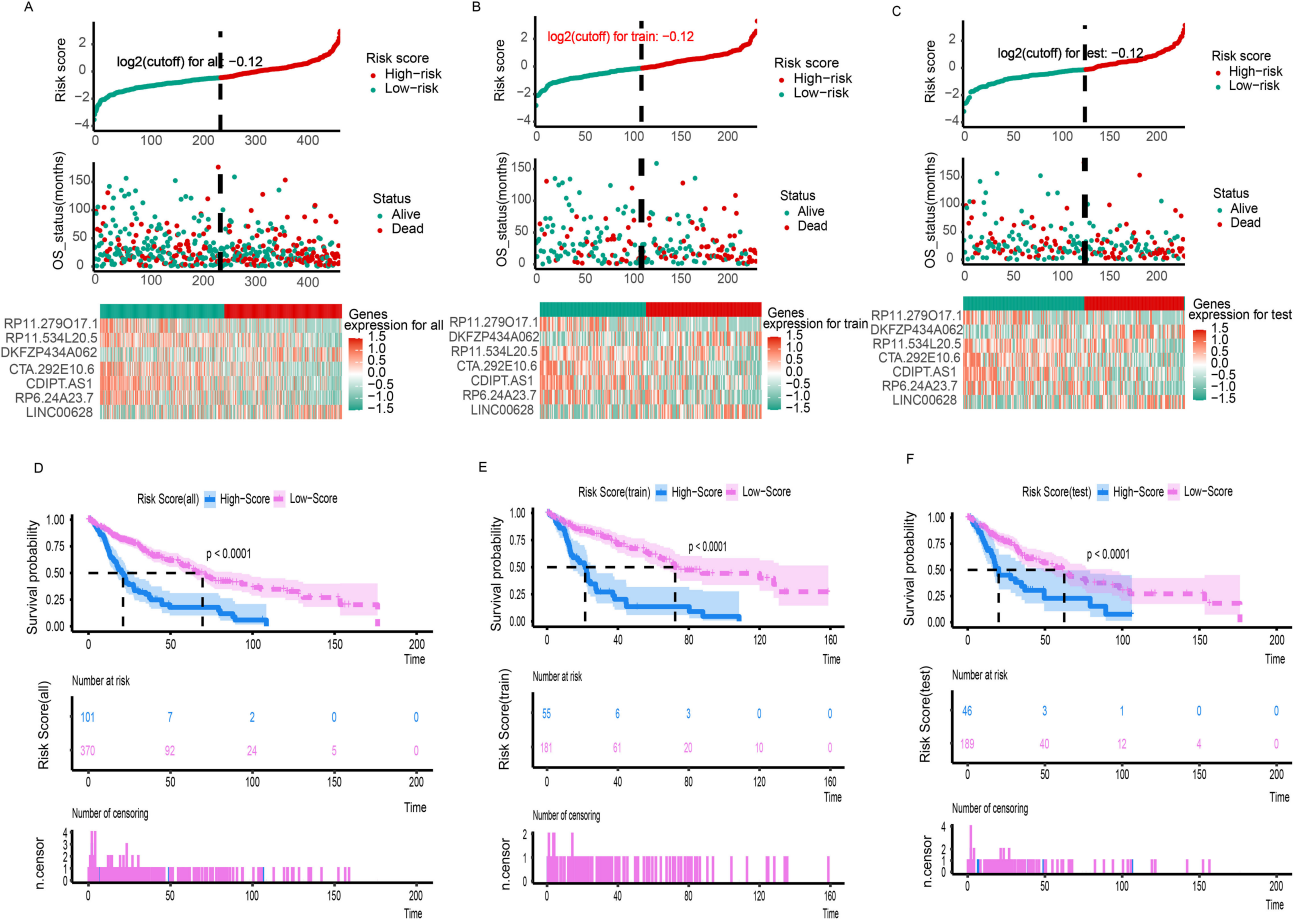
The risk score distribution, duration and survival statues of LC patients and heatmaps of the seven-gene signature relative expression in the total TCGA cohort **(A)**, training TCGA cohort **(B)**, and testing TCGA cohort **(C)**. Kaplan-Meier analysis of the low‐ and high-risk group patients in the total TCGA cohort **(D)**, training TCGA cohort **(E)**, and testing TCGA cohort **(F)**.

**Figure 2:**
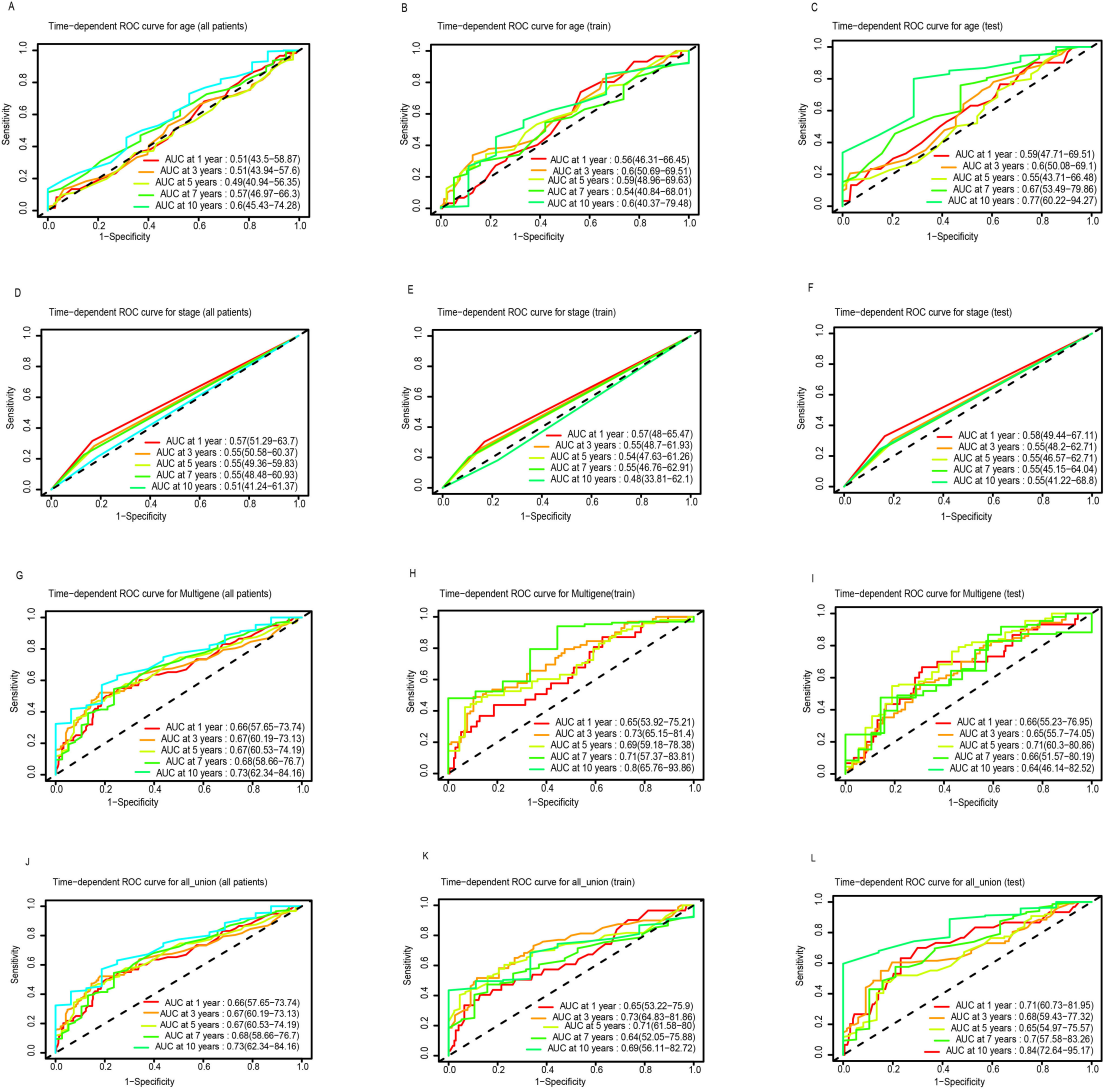
ROC curve analysis of age, stage, multigene and all union index according to the 1, 3, 5, 7, and 10-year survival of the area under the AUC value in the total TGCA cohort **(A, D, G, J)**, training TGCA cohort **(B, E, H, K)**, and testing TGCA **(C, F, I, L)**.

A total of 471 LUSC patients were randomly divided into a training dataset (n = 236) and a testing dataset (n = 235). Multivariate Cox regression was initially used to identify prognostic LncRNAs from the training set. This analysis revealed a significant association between the OS of LUSC patients and seven specific LncRNAs were RP11.279O17.1, DKFZP434A062, RP11.534L20.5, CTA.292E10.6, CDIPT.AS1, RP6.24A23.7, and LINC00628. A 7-LncRNA risk signature was constructed by linearly combining the expression levels of these seven LncRNAs, weighted by their respective Cox regression coefficients. The heatmap (**Figure 1B**) illustrates the relative expression levels of the seven prognostic LncRNAs, sorted according to their risk scores. Patients were then stratified into low- and high-risk groups based on an optimal cutoff for their risk scores, as shown in **Figure 1B**. Furthermore, the distribution of risk scores for each patient was visually represented, demonstrating that the mean survival time of high-risk patients was significantly shorter than that of low-risk patients, with a higher mortality rate observed in the high-risk group (**Figure 1B**). Kaplan-Meier survival analysis showed that patients in the high-risk group had significantly poorer prognosis compared to those in the low-risk group (**Figure 1E**). Additionally, the AUCs for 1-, 3-, 5-, 7-, and 10-year OS in the training cohort were 0.65, 0.73, 0.69, 0.71, and 0.80, respectively (**Figure 2H**).

### Validation of the seven-LncRNA signature in the testing set and full dataset

The robustness of the 7-LncRNA signature was further validated in both the testing set and the entire cohort. As shown in **Figure 1C**, similar to the training cohort, the same risk score formula effectively stratified patients into high- and low-risk groups in the testing cohort using a cutoff of -0.12 (log2-transformed). A significant survival difference was observed between these two groups. Kaplan-Meier survival analysis confirmed that the high-risk group in the testing cohort had a significantly worse prognosis compared to the low-risk group (P < 0.001; **Figure 1F**). Consistent results were obtained when analyzing the entire TCGA dataset of 471 patients (**Figures 1A, D**). The AUCs for 1-, 3-, 5-, 7-, and 10-year OS in the testing cohort were 0.71, 0.68, 0.65, 0.70, and 0.84, respectively (**Figure 2L**). For the overall cohort, the AUCs for OS at these time points ranged from 0.66 to 0.73 (**Figures 2J, K**). Additionally, clinical factors such as age (**Figures 2A, C**), stage (**Figures 2D, F**), and multigene expression (**Figures 2G, I**) were also analyzed across risk groups. These findings are consistent with previous studies, supporting the 7-LncRNA signature as a robust prognostic indicator for LUSC patients.

### Development and validation of a predictive nomogram

**Figure 3:**
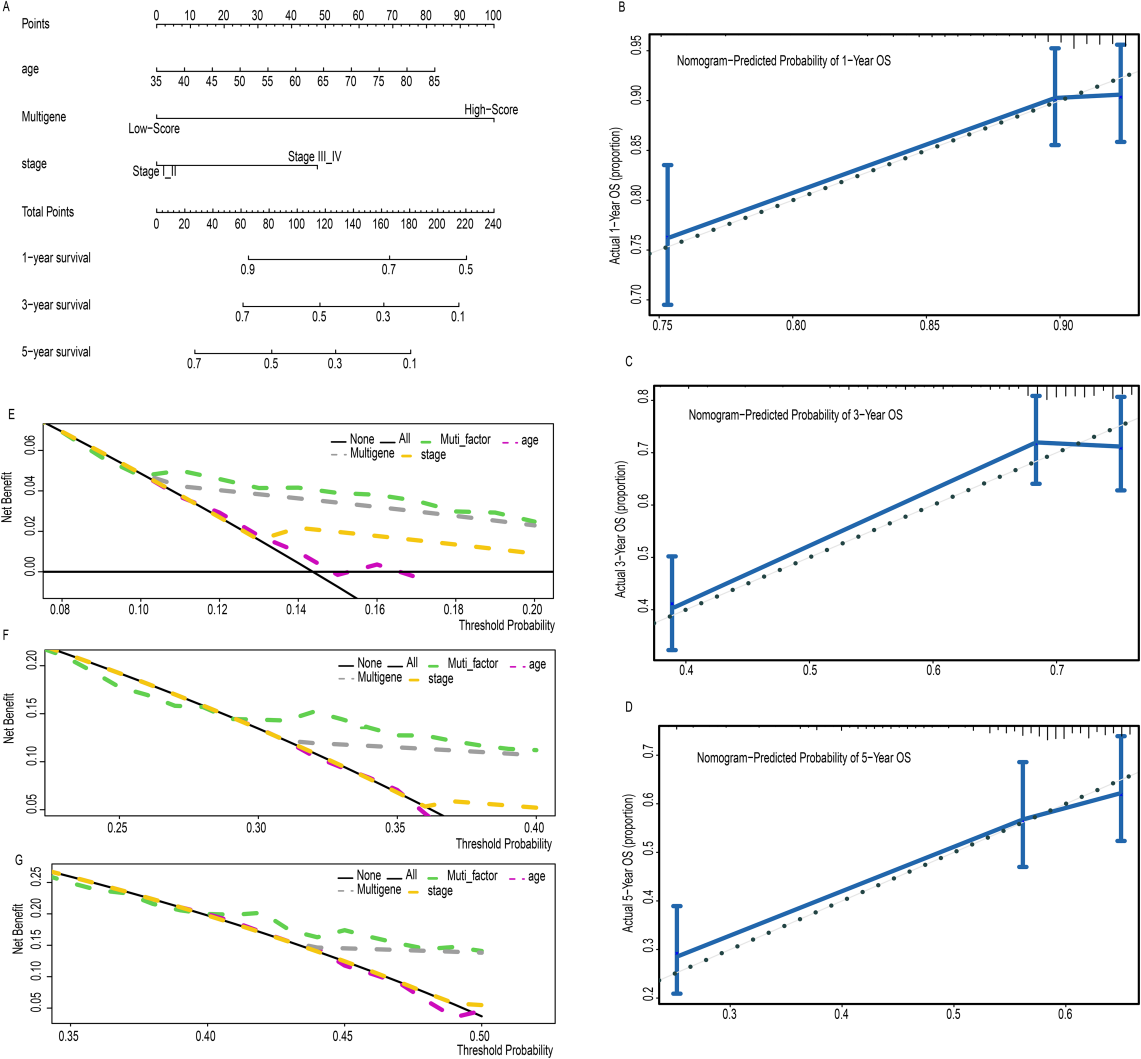
A prognostic nomogram predicting 1-, 3-, and 5- year OS of LC **(A)**. Calibration plots of the nomogram for predicting the proportion of patients with 1-, 3-, or 5-year OS **(B-D)**. Decision curve analysis of nomogram predicting 1-, 3-, and 5- year OS of LC comparing the age, stage and multigene **(E-G)**.

To improve the accuracy of survival predictions for LUSC patients, a prognostic nomogram was developed based on clinical data from 471 LUSC patients, integrating the risk score derived from clinical factors such as age, multigene expression, and tumor stage (**Figure 3A**). Calibration plots demonstrated that the nomogram performed well in predicting 1-, 3-, and 5-year OS for LUSC patients (**Figures 3B–D**). Decision curve analysis showed that the nomogram outperformed other models at various threshold probabilities (**Figures 3E–G**). These results suggest that the prognostic nomogram is highly effective in predicting 1-, 3-, and 5-year survival outcomes for LUSC patients.

### Independence of the LncRNA signature for survival prediction and subgroup analysis

**Figure 4:**
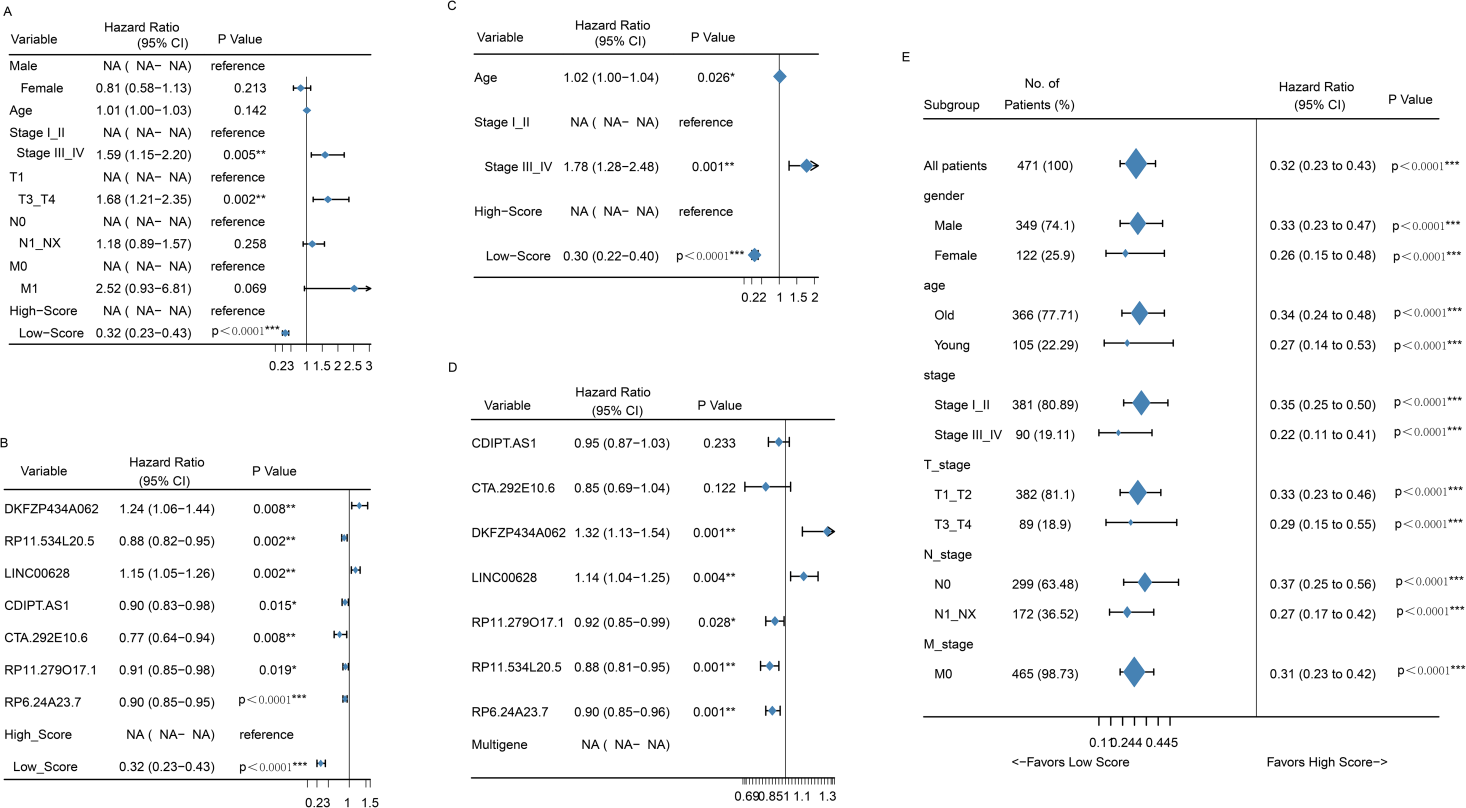
Forrest plot of the univariate Cox regression analysis OS of clinical factors **(A)** and seven-gene signatures **(B)**. Forrest plot of the multivariate Cox regression analysis OS of clinical factors **(C)** and seven-gene signatures **(D)**. Forrest plot of the univariate Cox regression analysis OS of risk score group in subgroup of clinical factors **(E)**. *p<0.05; **<0.01; ***<0.0001.

Univariate and multivariate Cox regression analyses were conducted to evaluate whether the prognostic value of the 7-LncRNA signature was independent of other clinical factors. The results showed that both the risk group and the 7-LncRNA signature were independent prognostic indicators for LUSC patients in univariate analysis (**Figures 4A, B**). In multivariate analysis, after adjusting for clinical variables such as age and AJCC stage, the risk group and 7-LncRNA signature remained significant independent prognostic factors (**Figures 4C, D**). Furthermore, the prognostic efficacy of the 7-LncRNA signature was consistent across various subgroups in the entire cohort, stratified by age (**Supplementary Figure S1A**), gender (**Supplementary Figure S1B**), AJCC stage (**Supplementary Figure S1C**), and TNM grade (**Supplementary Figure S1D–F**).

### Potential biological roles of the LncRNA signature

**Figure 5:**
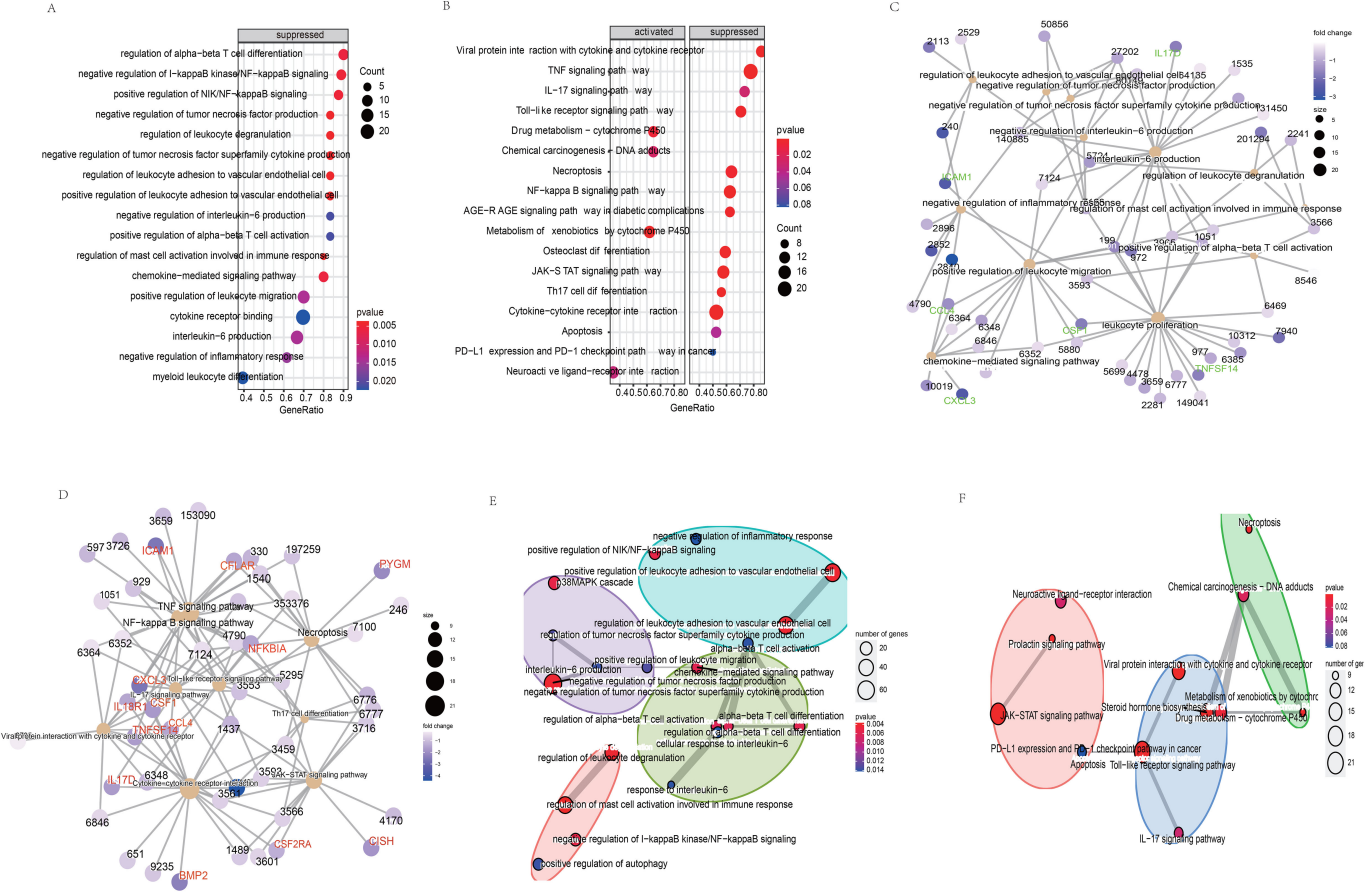
Biological functions **(A)** and the signaling pathways **(B)** of co-expressed genes related the model LncRNAs. The network regulation relationship between the co-expressed genes and biological functions **(C)**/the signaling pathways **(D)**. The network regulation relationship among the biological functions **(E)**/the signaling pathways **(F)**.

**Figure 6:**
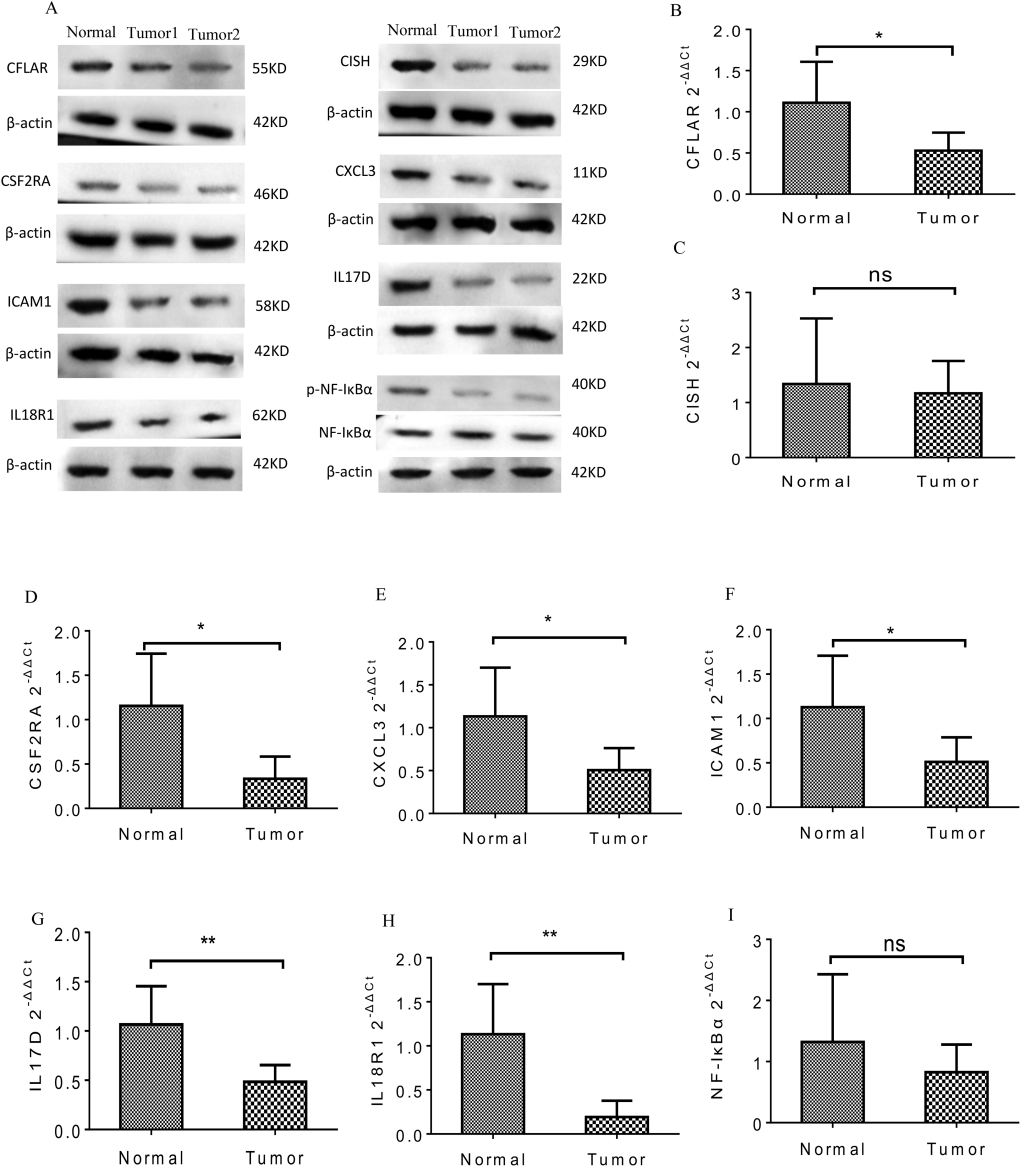
The PCR and WB analysis results of the eight genes in tumor tissues. **(A)** Protein expression of the eight genes was significantly decreased in the tumor groups. **(B-I)** mRNA expression of the eight genes was significantly inhibited in the tumor groups. *p<0.05; **<0.01; NS>0.05.

KEGG pathway analysis was performed on the protein-coding genes that were significantly associated with the model LncRNAs in TCGA-LUSC, using the entire human genome as the background. The results revealed that the prognostic LncRNAs were primarily enriched in pathways related to immune function, including Th17 cell differentiation, TNF signaling, NF-κB signaling, JAK-STAT signaling, Toll-like receptor signaling, and cytokine-cytokine receptor interaction (**Figure 5B**, P < 0.05). Moreover, the expression levels of the enriched genes were notably suppressed in LUSC patients. Gene Ontology (GO) enrichment analysis indicated that co-expressed genes were significantly downregulated and enriched in immune-related GO terms, such as mast cell activation involved in immune response, negative regulation of TNF production, regulation of leukocyte degranulation, and negative regulation of tumor necrosis factor superfamily cytokine production (**Figure 5A**, P < 0.05). These findings were further supported by cnetplot analyses for KEGG and GO (**Figures 5C, D**), and emapplot analysis revealed network interactions among key KEGG pathways and GO terms (**Figures 5E, F**). The expression levels of 14 target genes (BMP2, CCL4, CFLAR, CISH, CSF1, CSF2RA, CSF3, CXCL3, ICAM1, IL17D, IL18R1, NFKBIA, PYGM, TNFSF14) associated with tumor immune tolerance were significantly suppressed (**Figure 5D**). PCR and western blot analyses confirmed the downregulation of eight of these genes, yielding consistent results (**Figure 6**).

## Discussion

Lung cancer remains the leading cause of cancer-related mortality worldwide, yet treatment options continue to be limited. The clinical efficacy of available treatments is hindered by delayed diagnosis, limited therapeutic approaches, and the emergence of relapse and resistance (15). LUSC, a subtype of NSCLC, accounts for nearly 40% of all lung cancer cases. Early detection and timely intervention in LUSC can significantly improve patient prognosis, alleviating both the financial burden on patients and enhancing their overall quality of life (16). For decades, cancer research focused predominantly on protein-coding genes (17). However, recent studies have shifted toward exploring the role of non-coding RNAs (ncRNAs) in cancer, including microRNAs (miRNAs), long non-coding RNAs (LncRNAs), circular RNAs (circRNAs), and PIWI-interacting RNAs (piRNAs). These studies have highlighted the crucial regulatory roles of ncRNAs in cancer development and progression, broadening our understanding beyond protein-coding genes (18, 19). Chen et al. and Zhou et al. established a distinct panel of LncRNAs with significant diagnostic value for predicting the prognosis of LUAD (20, 21). Zhang et al. developed a prognostic model for LUSC patients based on nine specific LncRNAs (AC013457.1, AC124067.2, AP001189.1, AP002360.1, BANCR, LINC00519, LINC01807, MIR3945HG, FAM83A−AS1, and POU6F2−AS2) (22). However, research on prognostic biomarkers for LUSC patients remains limited.

In the present study, we further investigated the role of LncRNAs in LUSC and identified seven previously unstudied LncRNAs significantly associated with the OS of LUSC patients. A significant survival difference was observed between the low-risk and high-risk groups, stratified by the risk score derived from our model in the training set. In addition, mean survival time, mortality rates, and overall prognosis varied significantly between these groups (**Figure 1**). These findings were validated in the testing dataset and across the entire cohort, yielding consistent results that have not been previously reported (22). We assessed the prognostic ability of our model using ROC curve analysis. Although the prognostic value of the AUC is modest, there is currently no better alternative for prediction. Relevant studies have also used AUC values to predict overall survival (OS), demonstrating its strong predictive ability (23, 24). In our study, the AUCs for 1-, 3-, 5-, 7-, and 10-year OS in the training group were 0.65, 0.73, 0.69, 0.71, and 0.80, respectively (**Figure 2H**). Notably, the AUCs for 1- and 5-year OS were consistent with those reported in previous studies, further confirming the reliability of our results (23, 24). Moreover, our study offers more detailed information and greater accuracy than the single AUC value of 0.65 for 3-year survival reported by Zhang et al. (22). Notably, our prognostic model outperformed other clinical variables, including TNM stage (AUCs of 0.57, 0.55, 0.54, 0.55, and 0.40) and age (AUCs of 0.56, 0.60, 0.59, 0.54, and 0.60), across all time points in predicting the prognosis of LUSC patients. A prognostic nomogram was constructed, demonstrating excellent accuracy in predicting 1-, 3-, and 5-year OS, an achievement not previously reported in the literature (**Figure 3**). Multivariate Cox regression analysis confirmed that the 7-LncRNA signature remained an independent predictor of OS in LUSC patients (**Figure 4**). Furthermore, subgroup analysis confirmed the strong predictive ability of the risk score for OS across various LUSC subpopulations, stratified by age, gender, tumor stage, and other clinical features (**Supplementary Figure S1**). These results suggest that the 7-LncRNA signature is a robust prognostic marker for LUSC that remains independent of other clinical variables.

To explore the underlying biological mechanisms of tumor immune inhibition in LUSC, we performed GO and KEGG enrichment analyses based on the 7-LncRNA model. Th17 cells, along with their associated cytokines, are implicated in immune responses across various tumors (25). IFN-γ and IL-17 stimulate Th17 cell differentiation, leading to the production of CXCL9 and CXCL10, which recruit Th1 and NK cells to the tumor microenvironment, enhancing antitumor immune responses (26). Interestingly, our results indicated that the expression of protein-coding genes involved in Th17 cell differentiation (**Figure 5B**) was significantly suppressed in LUSC patients (**Figure 5D**). NF-κB plays a key role in immune cell function, promoting inflammation by inducing the expression of cytokines and chemokines, which inhibit tumor growth (27). It has also been implicated in tumorigenesis (28). The activation of TNF-α and IL-1, which stimulate NF-κB through their receptors, enhances innate immune responses and promotes tumor cell apoptosis (29, 30). TNF-α exhibits antineoplastic properties (31), and its receptors are significantly downregulated in high-stage NSCLC. Additionally, STAT3 and STAT5, members of the STAT family, are implicated in tumor initiation and progression (32). It has been shown that STAT3 inactivation reduces TNF-α expression, leading to a loss of its ability to bind the TNF-α promoter (33, 34). Our findings (**Figures 5**, **6**) demonstrated significant downregulation of genes associated with the TNF pathway (NFKBIA, CSF1, ICAM1, CXCL3), the NF-κB pathway (CCL4, CFLAR, NFKBIA, TNFSF14, ICAM1, CXCL3), JAK-STAT signaling (CSF2RA, CISH, CSF3), Toll-like receptor signaling (CCL4, NFKBIA), and Th17 cell differentiation (NFKBIA), further supporting the involvement of these pathways in LUSC (35–37).

**Figure 7:**
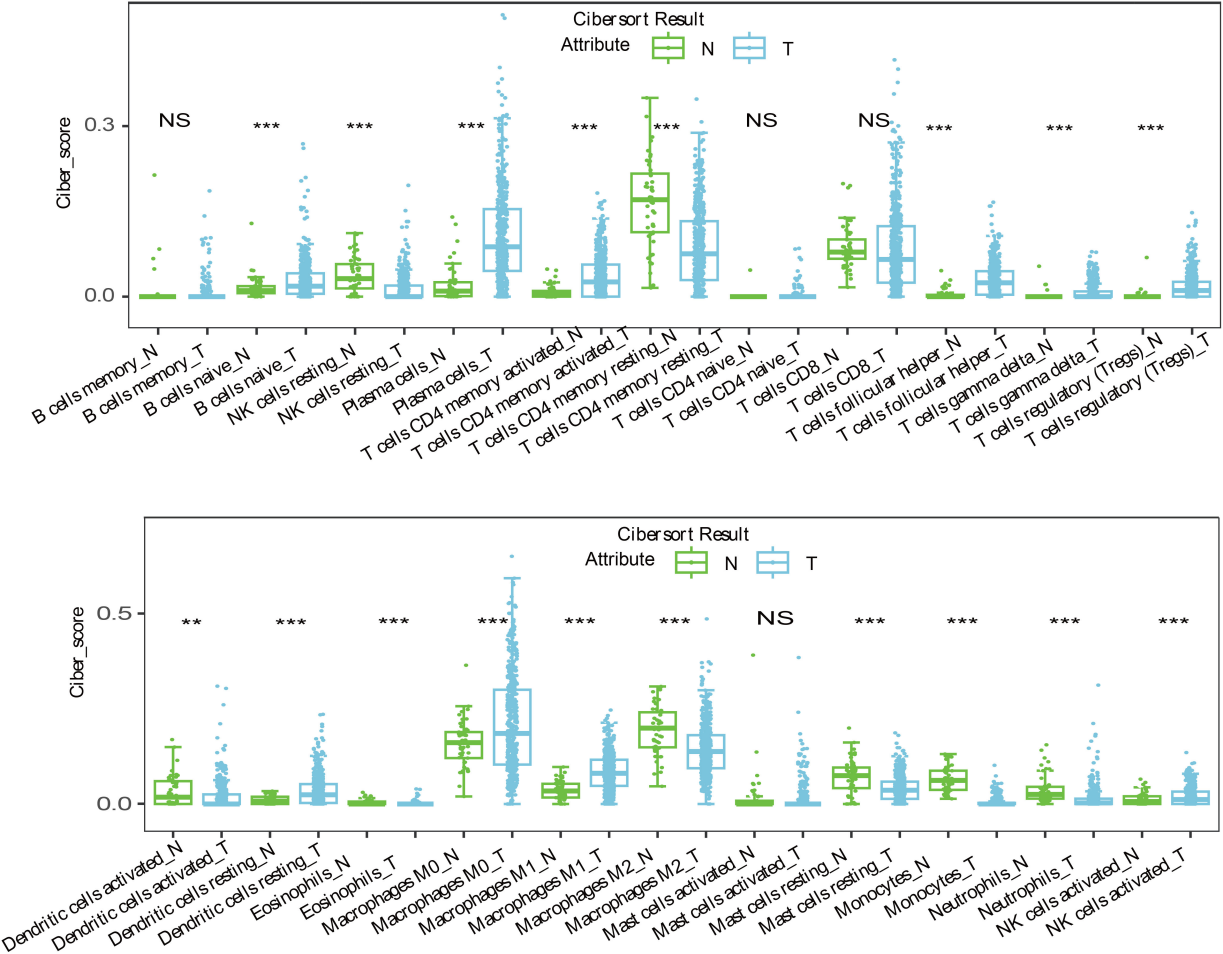
The immune infiltration reaction including T cells, B cells, NK cells and monocytes were prominent active in LUSC patients. **<0.01; ***<0.0001; NS>0.05.

Immune escape is a critical mechanism in tumorigenesis, and recent studies have shown that LncRNAs like SNHG12 facilitate immune evasion in NSCLC by interacting with HuR to increase PD-L1 and USP8 levels (38). Our study revealed that 14 target genes associated with immune tolerance were significantly suppressed in LUSC and were linked to the 7-LncRNAs of our risk model (**Figures 5**, **6**). Despite this, immune infiltration, including T cells, B cells, NK cells, and monocytes, was prominently activated in LUSC patients (**Figure 7**). While numerous studies have reported immune infiltration in tumors such as breast cancer and glioblastoma multiforme (39, 40), not all immune infiltrates exert antitumor effects (41, 42). The mechanisms by which the seven prognostic LncRNAs contribute to immune escape in LUSC remain unclear and warrant further investigation.

There are several limitations to this study. First, the 7-LncRNA signature was derived from a relatively small cohort of 236 patients. Second, while bioinformatics analyses provided insights into the potential functions of the LncRNAs, the exact molecular mechanisms remain unclear and require further validation through experimental studies. Third, due to insufficient data, we were unable to assess the impact of treatment strategies or medications on patient outcomes in LUSC. In conclusion, we have identified seven LncRNA biomarkers that can effectively predict OS in LUSC patients, providing valuable insights for prognostic prediction in this patient population.

## Data Availability

The raw data supporting the conclusions of this article will be made available by the authors, without undue reservation.

## Supplementary material

Supplementary Figure 1 Kaplan-Meier survival analysis of risk score group in subgroup of clinical factors. **(A)** age; **(B)** gender; **(C)** TNM stage; **(D)** N stage; **(E)** M stage; **(F)** T stage.
