# Supplementary figures and images for: A seven-LncRNA signature for prognosis prediction of patients with lung squamous cell carcinoma through tumor immune escape
Source: Front Oncol. 2025 Mar 24;15:1511564. doi: 10.3389/fonc.2025.1511564 (PMC11973350; doi:10.3389/fonc.2025.1511564)

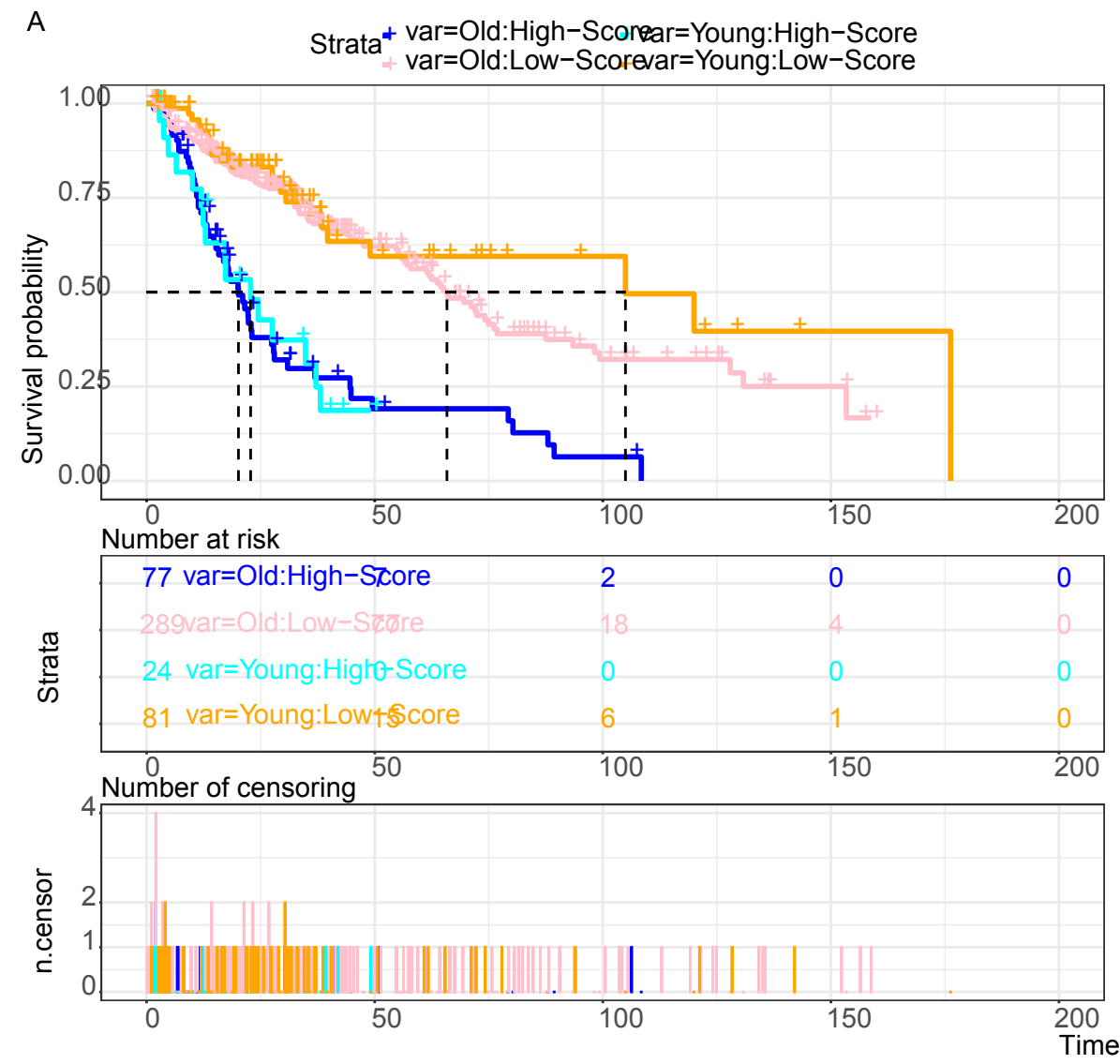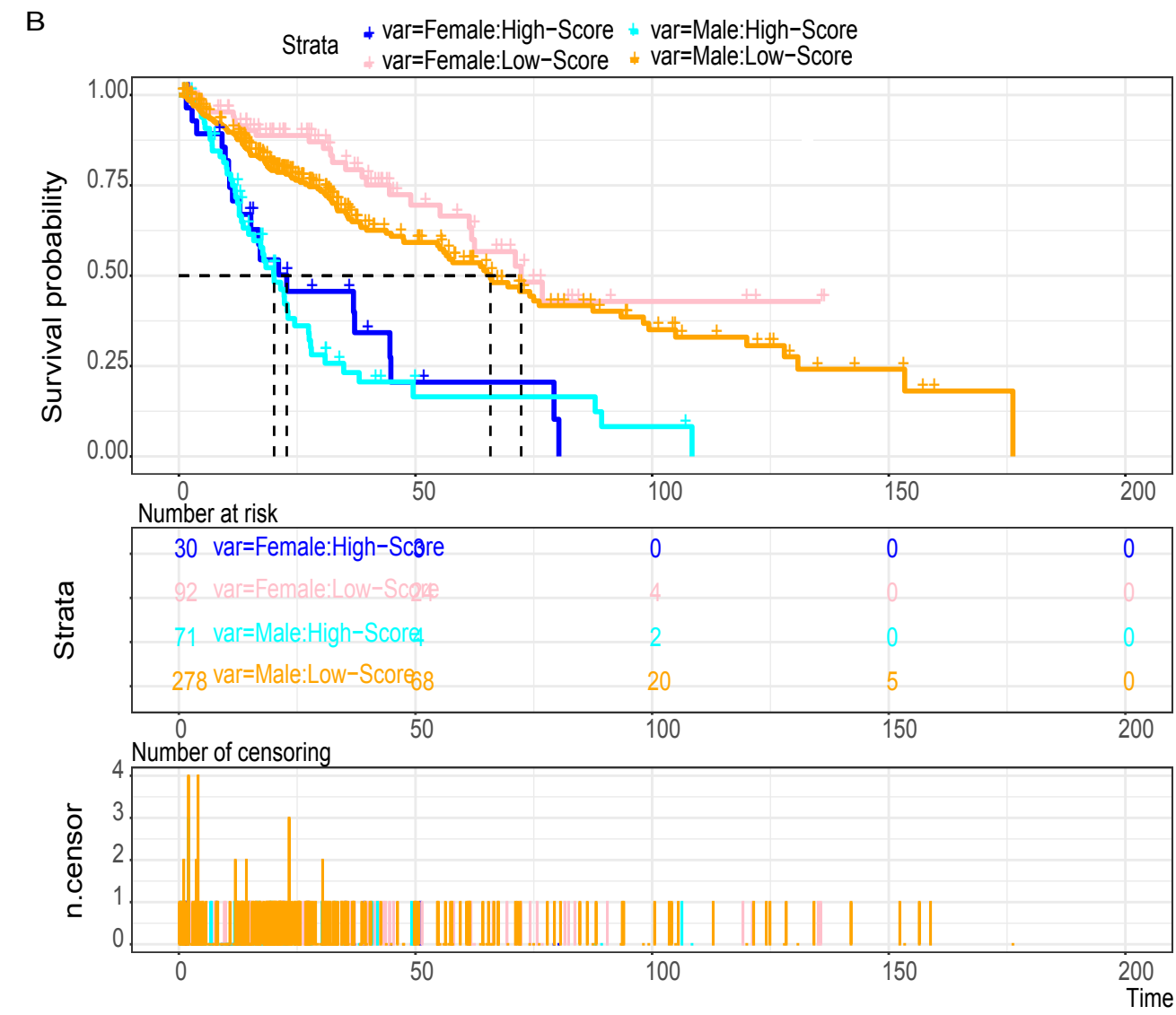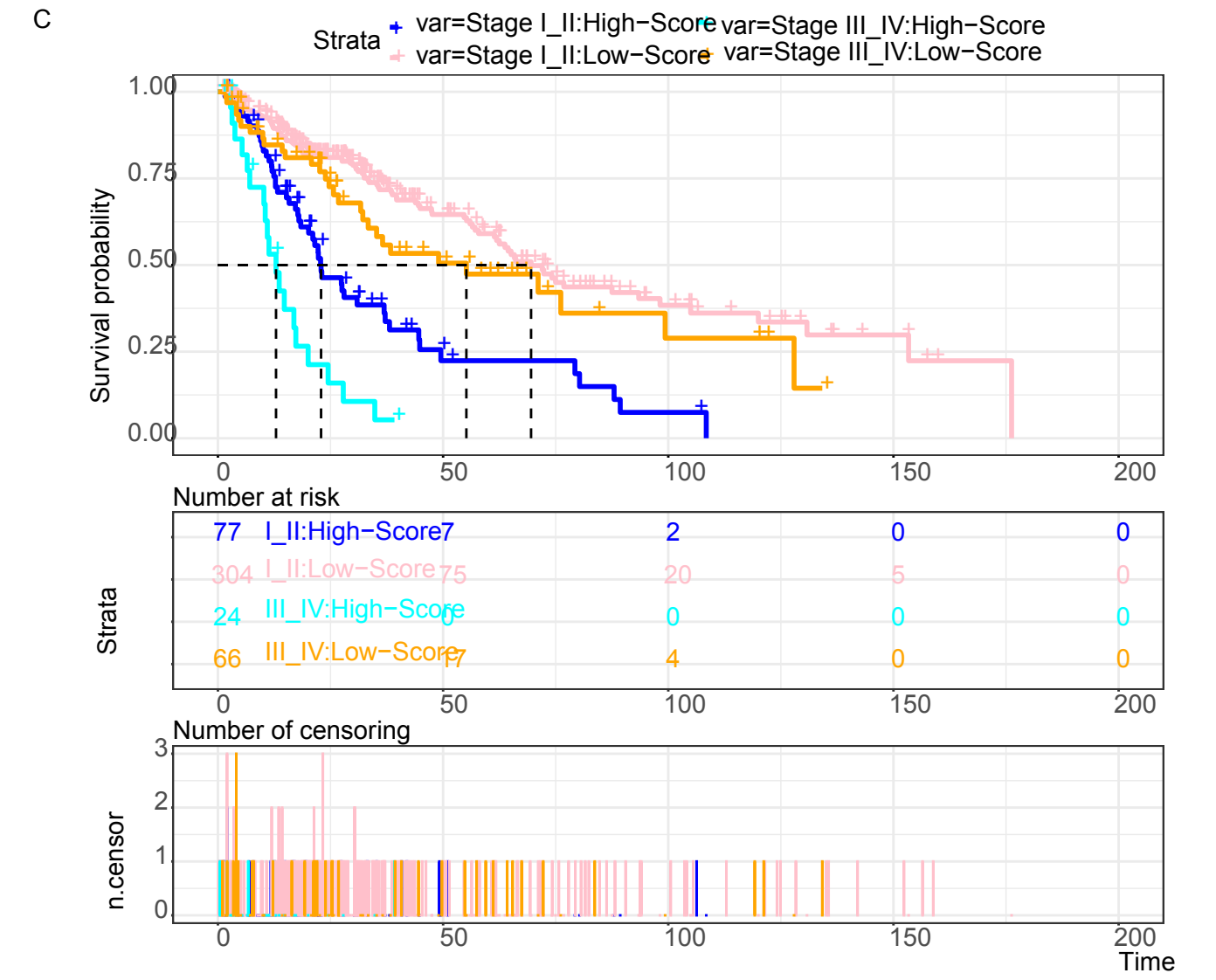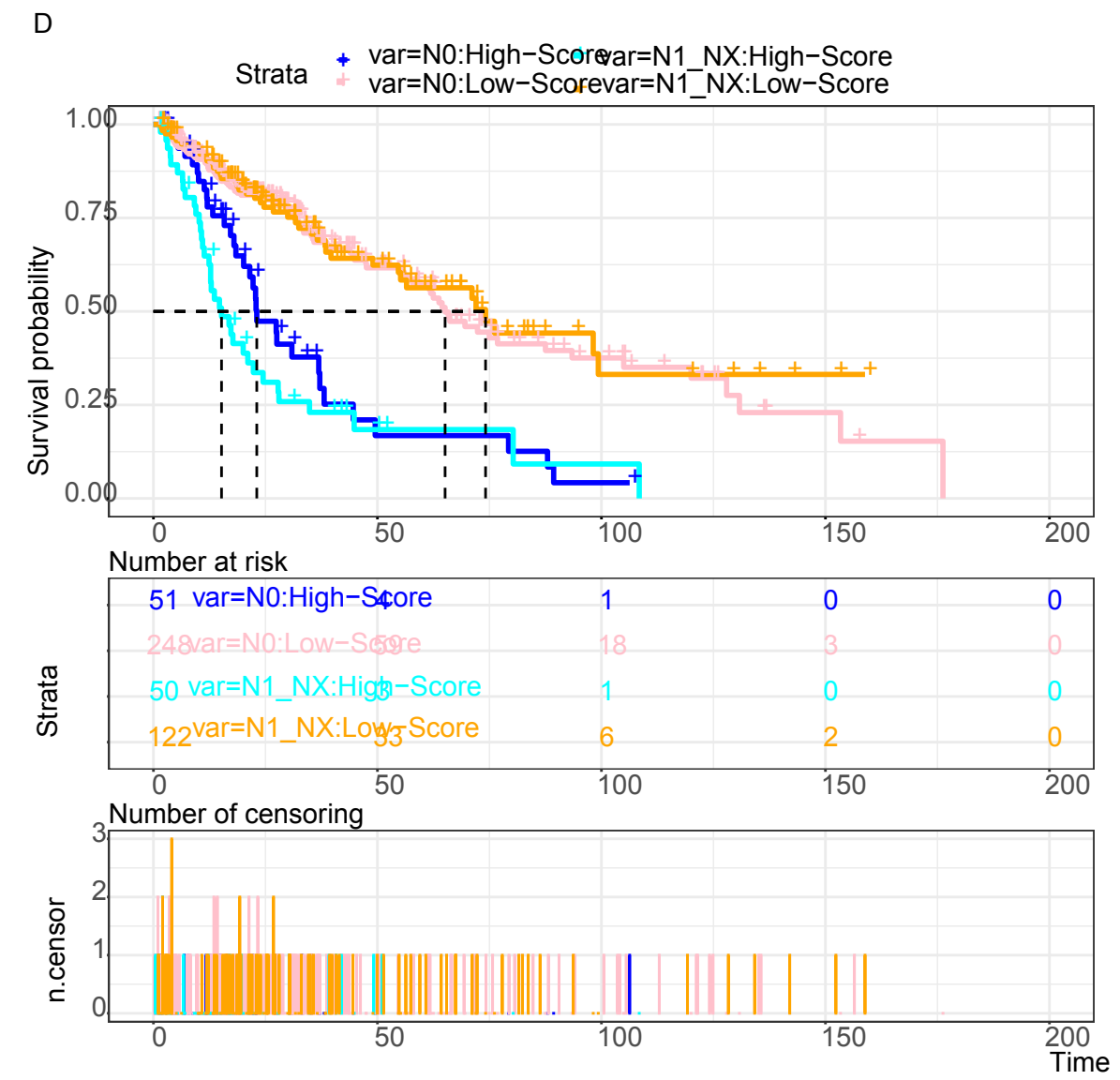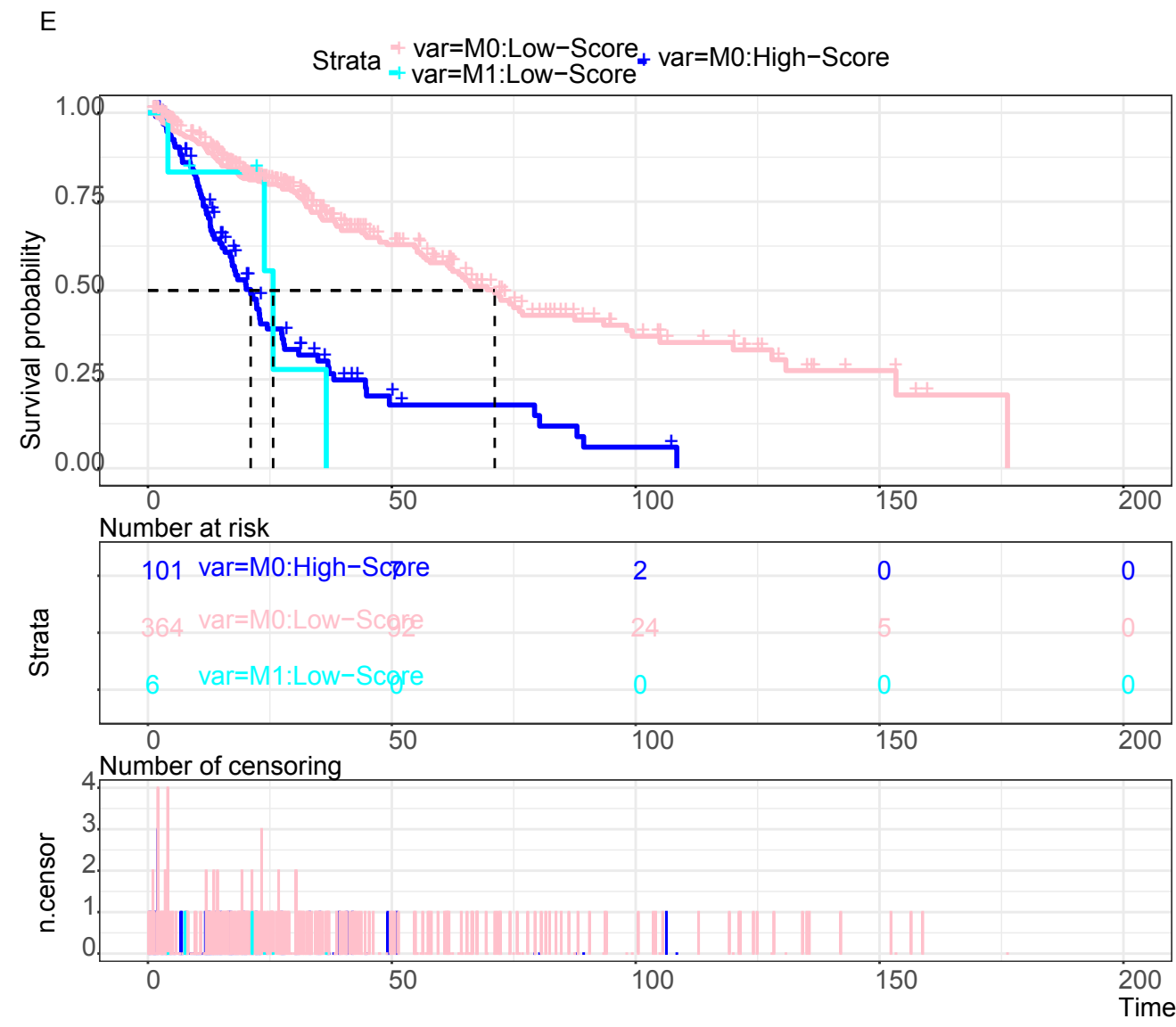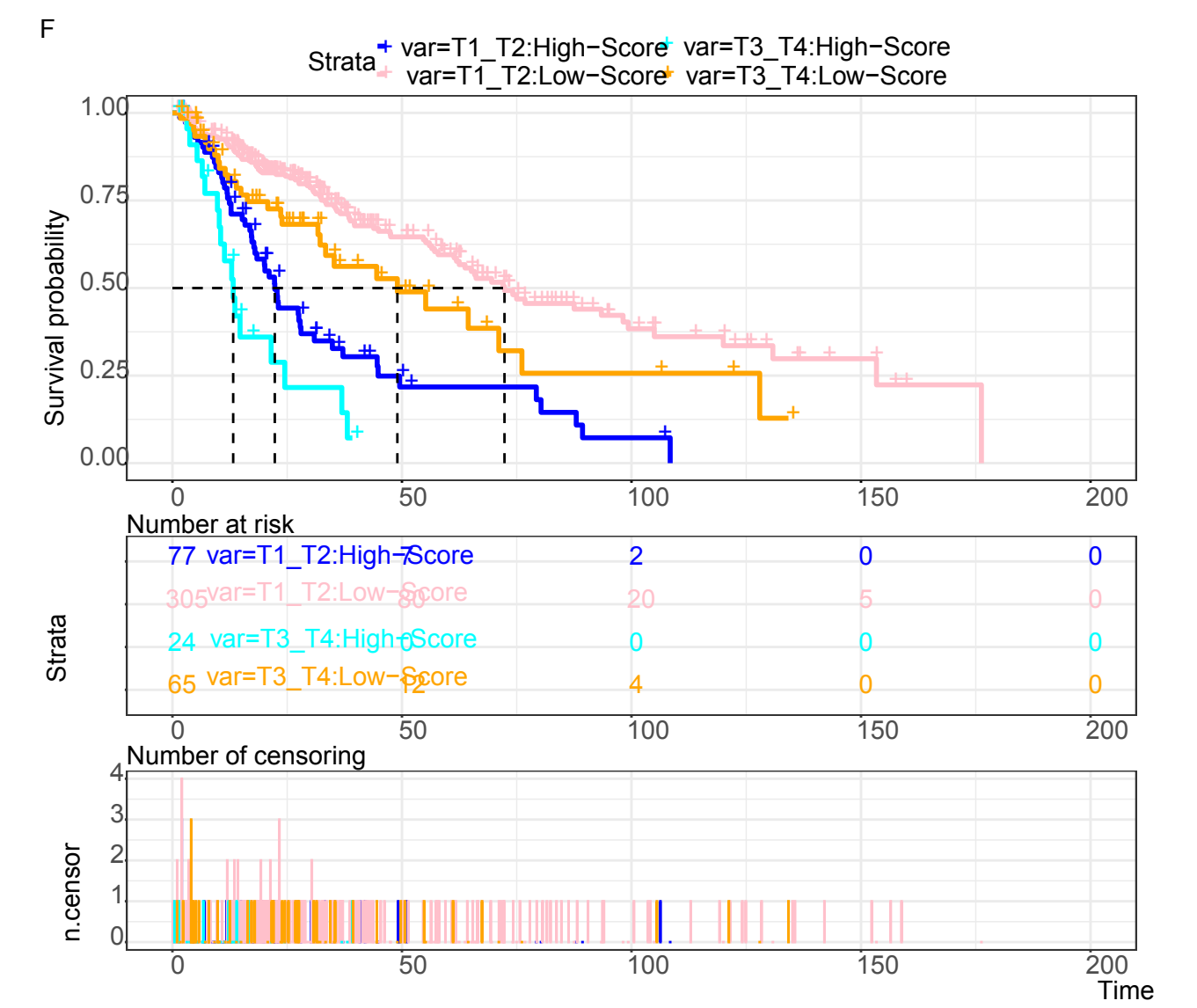

Supplement: Supplementary Figure 1 — Kaplan-Meier survival analysis of risk score group in subgroup of clinical factors. (A) age; (B) gender; (C) TNM stage; (D) N stage; (E) M stage; (F) T stage. [file Image1.pdf]
